# How Cognition and Motivation “Freeze” the Motor Behavior in Parkinson’s Disease

**DOI:** 10.3389/fnins.2019.01302

**Published:** 2019-12-06

**Authors:** Paola Ortelli, Davide Ferrazzoli, Veronica Cian, Marianna Zarucchi, Grazia Palamara, Alessandro Giobbia, Giuseppe Frazzitta, Roberto Maestri, Margherita Canesi

**Affiliations:** ^1^Department of Parkinson’s Disease, Movement Disorders and Brain Injury Rehabilitation, “Moriggia-Pelascini” Hospital, Como, Italy; ^2^School of Specialization in Neuropsychology, Vita-Salute San Raffaele University, Milan, Italy; ^3^MIRT ParkProject, Livorno, Italy; ^4^Department of Biomedical Engineering, Istituti Clinici Scientifici Maugeri IRCCS SpA SB, Pavia, Italy

**Keywords:** freezing of gait, Parkinson’s disease, cognition, motivation, motor behavior

## Abstract

**Objective:**

Freezing of gait (FoG) is a debilitating problem in patients with PD. The multifactorial pathogenesis of FoG remains poorly understood. We aimed to find which factors are most strongly associated with the occurrence of FoG.

**Methods:**

Three hundred five PD patients were enrolled and subdivided according to the presence (FoG +, *n* = 128) or absence (FoG-, *n* = 177) of FoG. Several clinical, functional, and neuropsychological data were collected and compared between groups. The association between the probability of presence of FoG and possible explanatory variables was assessed by logistic regression analysis.

**Results:**

FoG + patients were younger at the diagnosis (*p* = 0.04), and their mean daily dose of dopaminergic drugs (*p* < 0.0001) was higher in comparison with FoG- patients. FoG + patients get worse in Frontal Assessment Battery (*p* = 0.005), had higher scores in Apathy Evaluation Scale (*p* = 0.03), and were much more impaired on Wisconsin Card Sorting Test (WCST) (*p* = 0.018), Trail Making Test A (*p* = 0.0013), and Ray Auditory Verbal Learning Test (*p* = 0.012). Levodopa equivalent dose, age (direct), age at disease onset (inverse), and WCST were significant predictors of FoG (*p* = 0.01, *p* = 0.0025, *p* = 0.0016, and *p* = 0.029, respectively).

**Conclusion:**

FoG + patients show more deficits in executive functions and in motivation. The main explanatory variables of FoG occurrence are levodopa equivalent dose, age, age at disease onset, and WCST. These data suggest that a specific involvement of frontal cortical circuits in PD is responsible for certain cognitive–behavioral alterations related to the occurrence of FoG.

## Introduction

Freezing of gait (FoG) is a mysterious and debilitating symptom of Parkinson’s disease (PD) causing reduced mobility, falls and poor quality of life (Giladi and Nieuwboer, 2008; Rahman et al., 2008). It is defined as a “brief, episodic absence or marked reduction of forward progression of the feet despite the intention to walk” (Nutt et al., 2011). It is most frequent in the later stages of PD (70%) (López et al., 2010) but can also occur in 26% of early-stage patients (Giladi et al., 2001). Despite freezing is commonly observed while walking, it could occur in other behavioral, non-gait conditions, including foot tapping, speech, and handwriting (Vercruysse et al., 2014), as a form of sudden paroxysmal arrests or movement breakdown (Lewis and Barker, 2009; Vercruysse et al., 2014). FoG has been related to alterations in cortico-basal ganglia information processing or to disruption of cortical- and cerebellar-brainstem connections (Shine et al., 2013; Vercruysse et al., 2015).

The linking between FoG and dopaminergic drugs is fascinating: the term “freezing” appeared relatively late in the literature (Garcia-Ruiz, 2011), after the introduction of levodopa as therapy for PD (Ambani and Van Woert, 1973). While the early appearance of FoG is considered a “red flag” to diagnose forms of atypical parkinsonism, its occurrence during the PD course usually represents a hypokinetic “off” phenomenon (Snijders et al., 2016). Despite FoG is often levodopa responsive, it could be also paradoxically induced or worsened by dopaminergic medications (Devos et al., 2010; Espay et al., 2012). The pathophysiology of FoG and the relation with the pharmacological therapy raise many questions. Even though alterations of the dopaminergic system contribute to FoG, an involvement of other neurotransmitter pathways has been demonstrated to be deterministic for this phenomenon (Perez-Lloret and Barrantes, 2016). Specifically, a relation among the involvement of the cholinergic system, FoG, and cognitive impairment has been found in patients with PD (Bohnen et al., 2012; Xiao et al., 2017). Altered functioning of frontal neural circuits leading to executive dysfunctions could also play a central role in the physiopathology and the severity of FoG (Amboni et al., 2008; Ricciardi et al., 2014; Teramoto et al., 2014; Peterson et al., 2016). In a dopaminergic PET study, Bartels et al. (2006) showed a greater caudate nucleus denervation in patients with FoG compared with those without FoG. The prominent involvement of caudate nucleus could explain many cognitive aspects characterizing patients with FoG: indeed, the connections between the caudate nucleus and the prefrontal regions underline properly the executive functions, which are crucial for modulating and initiating behavioral responses relative to the environment (Leh et al., 2010; Leisman et al., 2014). Not surprisingly, it has been observed that specific cognitive demands (e.g., dual task) can trigger or exacerbate FoG episodes (Spildooren et al., 2010; Snijders et al., 2016; Gilat et al., 2018). Moreover, the knowledge of how the dynamic interactions of distributed brain areas operate in large-scale networks lead to the understanding that the executive network is subtended by both the dorsolateral prefrontal cortex and the posterior parietal cortex (Cavanna and Trimble, 2006; Bressler and Menon, 2010). Recently, the parietal lobe was supposed to contribute to FoG (Rubino et al., 2014; Brugger et al., 2015). A growing body of evidence indicates the posterior parietal cortex as an area involved in sensory integration, control of spatial aspects of motor behavior, visuo-spatial processes, and voluntary attention shifts between targets (Cavanna and Trimble, 2006). Rubino et al. (2014) found that, properly, a large cluster of gray matter volume reduction in the posterior parietal cortex contributes to the occurrence of FoG. Moreover, a metabolic reduction in parietal association areas and an increase in the cerebellar *vermis* and dentate nuclei are considered to be deterministic for cognitive dysfunctions in PD (Huang et al., 2007). Some authors concluded that visuospatial tests and deficits in visuospatial perception processing discriminate freezers from non-freezers and appear to be more strongly associated with the severity of FoG (Nantel et al., 2012; Heremans et al., 2013). Finally, several studies suggest that different cognitive deficits may contribute to various aspects of freezing in PD (Nantel et al., 2012): these observations support the “perceptual hypothesis” confirming the role of cortical structures, particularly those implicated in sensory integration, in the pathogenesis of FoG (Herman et al., 2014).

The role of affective disturbances is controversial: some authors found relations among anxiety, depression, and FoG (Giladi and Hausdorff, 2006; Ehgoetz Martens et al., 2014), while others (Suppa et al., 2017) did not find affective differences between freezers and non-freezers.

Finding which motor, cognitive, functional, pharmacological, and affective factors are associated with FoG and which ones predict its occurrence is fundamental to create a model of the physiopathology of this phenomenon in a therapeutic perspective. Clinical predictors of FoG have been already analyzed in previous studies, often with conflicting results. Giladi et al. (2001) found that longer disease duration, higher non-tremor score, the absence of tremor, and initial symptoms as a gait disorder can predict the development of FoG. In another study (Forsaa et al., 2015), while no associations were found between FoG and disease severity, the authors showed that the presence of motor fluctuations and higher levodopa dosages are independent risk factors for the development of FoG. In contrast, in a 3-year follow-up study, Zhang et al. (2016) did not find relations between levodopa treatment and future FoG occurrence. Most recently, it has been suggested that patients with onset in the lower limbs and presence of festination, falls, and hallucinations may be prone to develop FoG, which likely occurs with deterioration of both PD severity and visuospatial functions (Ou et al., 2018).

In this study, we analyze a model of explanatory variables to find which factors, in the clinical and neuropsychological spectrum of PD, are most strongly associated with the occurrence of FoG. Furthermore, by integrating our clinical results with literature data, we will hypothesize a possible explanation of this complex phenomenon.

## Materials and Methods

### Study Population

The study sample comprised consecutive PD patients who attended the Department of Parkinson’s Disease, Movement Disorders and Brain Injury Rehabilitation of “Moriggia-Pelascini” Hospital (Gravedona ed Uniti—Como, Italy) during a 2-year period (between January 2015 and December 2017). The diagnosis of PD was confirmed according to the United Kingdom Parkinson’s disease Brain Bank criteria (Hughes et al., 1992). All PD patients had a positive response to levodopa and did not have clinically significant brain lesions, as seen by brain radiological study (computerized axial tomography or magnetic resonance imaging). Exclusion criteria included unclear diagnosis, neurological diseases other than PD, atypical or secondary parkinsonisms, severe comorbidities that interfere with daily functioning, deep bran stimulation surgery, and inability to complete clinical assessments. Patients with FoG were identified using the freezing of gait questionnaire (FOG-Q) (Giladi et al., 2000) by asking them “do you feel that their feet were glued to the floor while walking, making a turn or when trying to initiate walking?” (item 1.3). This measure has previously been shown to be a reliable screening to identify “freezers” (Shine et al., 2012). If the patient did not understand the question, researchers showed the phenomenon mimicking a FoG episode (Giladi et al., 2009). Patients who answered affirmatively were subsequently administered the FOG-Q in full. A neurologist with experience in movement disorders checked the information obtained from patients with relatives, caregivers, and clinical observations to ensure accuracy of data. Accordingly, patients were classified as freezers (FoG + patients) and non-freezers (FoG- patients).

The study design and protocol were approved by the local Ethics Committee (“Comitato Etico Interaziendale delle Province di Lecco, Como e Sondrio”) and were in accordance with the code of Ethics of the World Medical Association (Declaration of Helsinki, 1967). A complete explanation of the study protocol was provided, and written informed consent was obtained from all participants before their participation in the study.

### Clinical Evaluation

A neurologist with experience in movement disorders completed the clinical assessment. We first recorded gender, age, age at disease onset, disease duration, and the side of motor symptoms predominance at onset. Patients were evaluated in the morning, 60–90 min after they have taking their first dopaminergic drug dose, in medication “on” state. Levodopa equivalent dose (LED) was calculated for all the dopamine replacement therapies that patients were taking, using a standardized formula (Tomlinson et al., 2010). The Unified Parkinson’s Disease Rating Scale (UPDRS) parts I to IV (Fahn and Elton, 1987), which have been shown to strongly correlate with the corresponding parts in the MDS-UPDRS (Goetz et al., 2008), were assessed to evaluate the patients’ clinical conditions and various motor and non-motor aspects related to PD. To grade the severity of symptoms, the Hoehn & Yahr staging (H&Y) (Hoehn and Yahr, 1967) was also adopted. FoG was assessed using FOG-Q (Giladi et al., 2000). The total FOG-Q score ranges from 0 to 24, with high total scores corresponding to severe FoG.

To assess the sense of well-being perceived by subjects, we evaluated the quality of life of patients with the Parkinson’s disease Questionnaire 39 (PDQ-39) (Peto et al., 1995).

### Functional Evaluation

A physiotherapist with experience in movement disorders completed the functional evaluation. Specifically, Six-Minute Walk Test (6MWT) (Guyatt et al., 1985), Timed Up and Go test (TUG) (Podsiadlo and Richardson, 1991), and Berg Balance Scale (BBS) (Berg et al., 1992) were adopted to estimate different functional aspects. Patients were evaluated in medication “on” state. 6MWT is used to measure the distance walked over 6 min, as a submaximal test of aerobic capacity/endurance. 6MWT has been shown to have excellent test–retest reliability for subjects with PD (Steffen and Seney, 2008). TUG is a simple test used to assess a person’s mobility; it requires both static and dynamic balance and is recognized as useful for evaluating the executive component of action (Morris et al., 2001). It uses the time that a person takes to rise from a chair, walk 3 m, turn around, walk back to the chair, and sit down. TUG has been shown to have excellent test–retest reliability for subjects with PD (Morris et al., 2001). BBS is designed to rate balance in sitting, standing, turning, and reaching forward. High test–retest reliability of scores for the BBS has been shown in people with parkinsonism (Steffen and Seney, 2008).

### Neuropsychological Assessment

The neuropsychological battery was administered by a trained neuropsychologist blinded to the patients’ condition. All patients were tested in the morning in a laboratory setting, with constant artificial lighting condition and in absence of auditory interferences, in medication “on” state.

The cognitive profile was studied by administering Mini Mental State Examination (Magni et al., 1996), Frontal Assessment Battery (FAB) (Dubois et al., 2000), Wisconsin Card Sorting Test (WCST) (Grant and Berg, 1948), Trail Making Test Part A and Part B (TMT A and B) (Tombaugh, 2004), Stroop Test (Stroop, 1935), Rey Auditory–Verbal Learning Test (RAVLT) (Shin et al., 2006), Rey–Osterrieth complex figure (ROCF) copy and delayed recall (Caffarra et al., 2002), and verbal fluency (Novelli et al., 1986). To assess the patients’ affective condition, we administered: State-Trait Anxiety Inventory (STAI) Y I–II (Spielberger et al., 1983), Beck Depression Inventory (BDI) (Beck et al., 1961), and Apathy Evaluation Scale (AES) (Marin et al., 1991). All scores were corrected for current Italian normative values.

### Statistical Analysis

Descriptive statistics are reported as mean ± standard deviation for continuous variables and as numbers (percentage frequency) for discrete variables. Between-group comparisons were carried out by unpaired *t*-test or by the chi-square test for continuous and categorical variables, respectively. The association between FOG-Q and clinical and functional variables in FoG + patients was assessed by correlation analysis (Pearson *R*).

The association between the probability of presence of FoG and possible explanatory variables was assessed by logistic regression. All statistical tests were two-tailed, and statistical significance was set at *p* < 0.05. All analyses were carried out using the SAS/STAT statistical package, release 9.4 (SAS Institute Inc., Cary, NC, United States).

## Results

The study sample comprised 305 PD patients. FoG was observed in 128 patients (42%). No difference in the prevalence of FoG occurrence was found with respect to the side of motor symptoms predominance at onset (FoG was present in 39 and 44% of patients with left- and right-side motor symptoms predominance, respectively, *p* = 0.39). FoG was present in 48% of male and in 33% of female patients (*p* = 0.011).

Table 1 reports demographic, clinical, functional, and neuropsychological variables for the overall population and stratified according to the presence of FoG.

**TABLE 1:** Demographic, clinical, functional, and neuropsychological variables for the overall population and for patients stratified according to presence of FoG.

	**Variable**	**All patients (*N* = 305)**	**FoG + Patients (*N* = 128)**	**FoG–Patients (*N* = 177)**	***P*-value**
Demographic	Male gender (%)	189 (62%)	90 (70%)	99 (56%)	0.011
	Age (years)	67.3 ± 9.4	68.3 ± 8.5	66.5 ± 9.9	0.10
	Age at disease onset (years)	56.9 ± 10.3	55.4 ± 9.7	58.0 ± 10.7	0.037
	Education (years)	10.3 ± 4.4	10.4 ± 4.3	10.3 ± 4.4	0.76
Clinical	Side of motor symptoms predominance at onset – Left side (%)	135 (44%)	53 (41%)	82 (46%)	0.39
	Disease duration (years)	10.5 ± 5.9	12.8 ± 5.5	8.8 ± 5.6	< 0.0001
	H&Y	2.7 ± 0.5	2.9 ± 0.5	2.6 ± 0.5	< 0.0001
	LED (mgEq/die)	735 ± 417	882 ± 410	629 ± 390	< 0.0001
	UPDRS Total Score	42.1 ± 11.4	46.9 ± 10.4	38.6 ± 10.9	< 0.0001
	UPDRS IV	4.1 ± 3.5	5.3 ± 3.5	3.2 ± 3.3	< 0.0001
	UPDRS III	20.1 ± 5.4	21.0 ± 5.4	19.4 ± 5.2	0.007
	UPDRS II	15.1 ± 5.2	17.5 ± 4.6	13.4 ± 4.9	< 0.0001
Functional	6MWT (m)	313 ± 115	298 ± 125	325 ± 106	0.048
	BBS	45.4 ± 9.3	44.5 ± 8.8	46.0 ± 9.5	0.18
	TUG (s)	14.4 ± 10.6	16.1 ± 12.9	13.1 ± 8.3	0.017
	FOGQ	15.0 ± 3.5	15.0 ± 3.5		
Qol	PDQ 39	46.4 ± 23.2	51.1 ± 23.2	42.4 ± 22.5	0.007
Psychological	STAI Y I	42.8 ± 6.3	42.7 ± 6.3	42.9 ± 6.3	0.82
	STAI Y II	43.0 ± 7.5	43.2 ± 6.7	42.9 ± 8.1	0.82
	BDI	6.8 ± 4.6	7.4 ± 5.1	6.3 ± 4.0	0.09
	AES	33.7 ± 8.5	35.3 ± 8.8	32.6 ± 8.0	0.032
Cognitive: Global Cognition	MMSE	26.6 ± 3.1	26.5 ± 3.3	26.6 ± 3.0	0.70
Global Executive Functions	FAB	13.5 ± 3.4	12.9 ± 3.7	14.0 ± 3.1	0.005
Planning, Problem	WCST	77.4 ± 34.7	83.9 ± 31.4	72.9 ± 36.2	0.017
Solving And Flexibility	% of impaired	(44%)	(53%)	(38%)	0.018
Executive Attention	TMT A	48.1 ± 48.5	56.4 ± 58.1	42.5 ± 40.1	0.039
	% of impaired	(10%)	(17%)	(5%)	0.001
Divided Attention	TMT B	130.2 ± 96.1	133.3 ± 98.6	128.3 ± 94.9	0.67
	% of impaired	(9%)	(10%)	(8%)	0.61
Attentional Switching	TMT B-A	91.8 ± 74.0	94.7 ± 73.5	90.0 ± 74.5	0.44
	% of impaired	(13%)	(11%)	(14%)	0.59
Suppression Of	STROOP Test	22.9 ± 15.0	24.0 ± 15.5	22.2 ± 14.7	0.42
Interference Capability	% of impaired	(14%)	(17%)	(12%)	0.27
Memory:	RAVLT	41.1 ± 8.9	40.2 ± 9.3	41.6 ± 8.7	0.19
Verbal Learning	% of impaired	(8%)	(14%)	(5%)	0.017
Visuoconstructional	ROCF COPY	28.3 ± 6.8	28.4 ± 7.0	28.0 ± 6.6	0.67
Abilities	% of impaired	(37%)	(61%)	(45%)	0.40
	ROCF delayed recall	14.1 ± 7.5	14.7 ± 8.4	13.3 ± 5.9	0.19
	% of impaired	(22%)	(23%)	(22%)	0.89
Verbal Fluency	Semantic cue	40.7 ± 10.2	40.5 ± 10.3	41.0 ± 10.1	0.72
		(4%)	(2%)	(5%)	0.35
	Phonemic cue	31.7 ± 10.3	31.6 ± 10.5	31.8 ± 10.1	0.92
		(8%)	(6%)	(10%)	0.32

*Percentage of impaired performances in WCST, TMT A, TMT B, TMT B-A, Stroop Test, and RAVLT are reported (accordingly with normative data). H&Y, Hoehn and Yahr staging; LED, levodopa equivalent dose; UPDRS, Unified Parkinson’s Disease Rating Scale; 6MWT, Six-Minute Walk Test; BBS, Berg Balance Scale; TUG, Timed Up and Go test; FOGQ, Freezing of Gait Questionnaire; PDQ39, Parkinson’s Disease Questionnaire 39; STAI, State-Trait Anxiety Inventory; BDI, Beck Depression Inventory; AES, Apathy Evaluation Scale; MMSE, Mini Mental State Examination; FAB, Frontal Assessment Battery; WCST, Wisconsin Card Sorting Test; TMT, Trail Making Test; RAVLT, Rey Auditory–Verbal Learning Test; ROCF, Rey–Osterrieth Complex Figure.*

Mean age was not different between FoG + and FoG- patients. At the time of PD diagnosis, FoG + patients were younger than FoG- patients and were treated with higher doses of dopaminergic drugs (measured as LED). FoG + patients showed worse scores in all scales and tests: lower FAB score, higher TUG score (in seconds), lower distance walked at 6MWT, and worse UPDRS scores.

Health-related quality of life, as assessed by the PDQ-39, was worse in FoG +. AES score was higher in FoG + patients, while depressive symptoms (measured with BDI) did not differ in the two groups, as well as chronic and situational anxiety assessed with the STAI Y I–II, respectively.

Finally, FoG + patients showed a higher incidence of deficits in cognitive functions: impaired WCST (total score, 53 vs. 38%, *p* = 0.018) higher “non-perseverative errors” score (27 vs. 15%, *p* = 0.03), impaired TMT A (17 vs. 5%, *p* = 0.0013), and impaired RAVLT (14 vs. 5%, *p* = 0.017).

Considering FoG + patients, a significant association between FOG-Q and clinical variables was observed only with age (*r* = 0.176, *p* = 0.047), H&Y (*r* = 0.367, *p* < 0.0001), 6MWT (*r* = −0.265, *p* = 0.003), BBS (*r* = −0.187, *p* = 0.036), total UPDRS (*r* = 0.403, *p* < 0.0001), UPDRS II (*r* = 0.463, *p* < 0.0001), UPDRS III (*r* = 0.219, *p* = 0.013), UPDRS IV (*r* = 0.211, *p* = 0.017), FAB (*r* = −0.284, *p* = 0.001), and PDQ-39 (*r* = 0.268, *p* = 0.009).

The results of logistic regression analysis modeling the probability of FoG + using clinical, demographical, and neuropsychological explanatory variables are given in Table 2. Out of all candidate predictors considered, only LED, age (direct), age at disease onset (inverse), and WCST were significant predictors of FoG (*p* = 0.01, *p* = 0.0025, *p* = 0.0016, and *p* = 0.029, respectively).

**TABLE 2:** Results of logistic regression analysis modeling the probability of FoG + using clinical, demographical, and neuropsychological explanatory variables.

	**Wald chi-square**	***P-*value**	**Odds ratio**	**(95% CI)**	
LED (× 100)	6.6370	0.0100	1.146	1.033	1.272
Age	9.1309	0.0025	1.140	1.047	1.241
AGE AT DISEASE ONSET	9.9692	0.0016	0.884	0.819	0.954
GENDER	1.8911	0.1691	0.548	0.233	1.291
Side of motor symptoms onset	2.0239	0.1548	1.808	0.800	4.089
MMSE	0.4287	0.5126	1.073	0.869	1.326
FAB	0.0137	0.9069	0.990	0.831	1.179
WCST	4.7840	0.0287	2.558	1.103	5.935
TMT A	0.0487	0.8254	1.243	0.180	8.568
STROOP TEST	0.2006	0.6543	0.678	0.124	3.718
RAVLT	0.7534	0.3854	3.044	0.246	37.609
PDQ39	2.0274	0.1545	1.016	0.994	1.038
STAI Y I	1.1620	0.2810	0.959	0.889	1.035
STAI Y II	0.0924	0.7611	0.988	0.917	1.066
BDI	0.0804	0.7767	1.017	0.904	1.145
AES	0.3296	0.5659	1.018	0.959	1.081

*WCST, TMT A, Stroop Test, and RAVLT are considered as dichotomic variables (impaired versus normal performance). MMSE, Mini Mental State Examination; FAB, Frontal Assessment Battery; WCST, Wisconsin Card Sorting Test; TMT, Trail Making Test; RAVLT, Rey Auditory–Verbal Learning Test; PDQ39, Parkinson’s Disease Questionnaire 39; STAI, State-Trait Anxiety Inventory; BDI, Beck Depression Inventory; AES, Apathy Evaluation Scale.*

In particular, holding all other variables constant, a 100 mgEq/die increase in LED corresponded to a 15% increase in the probability of presenting FoG [95% confidence interval (95% CI), 3.3, 27.2%], 1 year increase in age corresponded to a 14% increase in the probability of presenting FoG (95% CI, 4.7, 24.1%), and 1 year increase in the age at disease onset corresponded to a 12% decrease in the probability of presenting FoG (95% CI, 18.1, 4.6%). Finally, an impaired WCST was associated with a 155% increase in the probability of presenting FoG (95% CI, 10.3, 593.5%).

## Discussion

This prospective study was aimed at exploring the relation between FoG occurrence and demographical, clinical, functional, and neuropsychological variables to provide effective models for treatment. For this purpose, we studied a model of explanatory variables and evaluated the involvement of the prefrontal cortex in FoG manifestation. In line with previous studies, the data we collected confirm that patients suffering from FoG present a more severe clinical condition (higher H&Y), worse motor symptoms (evaluated with UPDRS), more pronounced functional decline (evaluated with TUG and 6MWT), and, finally, worse quality of life, with respect to those without FoG. Moreover, we find that freezers, despite their worse clinical status, were taking a significantly higher dosage of dopaminergic drugs (measured as LED). In our sample, the majority of patients with FoG are male; they are older but their age at disease onset was consistently lower compared to patients without FoG. Finally, we observe much more cognitive deficits (worse scores/performances in FAB, WCST, TMT A, RAVLT) and a greater apathy in FoG + patients.

On this basis, we performed a data analysis modeling the probability of FoG occurrence based on clinical, demographic, and neuropsychological explanatory variables. The model shows that the older age at the moment of evaluation, a younger age at disease onset, higher intake of dopaminergic drugs, and pathological performances at WCST contribute to explain the FoG manifestation.

The cognitive profile we found in FoG + patients indicate that subjects suffering from this disturbance could present a prominent cortical involvement. In line with this hypothesis, previous studies highlighted that a greater cognitive impairment, particularly in frontal/executive domains, are associated with early onset of both Alzheimer’s disease and fronto-temporal dementia (Jacobs et al., 1994; Ye et al., 2015). Because of FoG + patients were younger at disease onset, this cortical involvement could be framed in terms of an “early” alteration. Therefore, it is conceivable that the long-term load of pathological cortical alterations expressed over the years is responsible for the development of those specific cognitive alterations that are related in turn to the development of FoG.

Following previous observations (Amboni et al., 2008), we conclude that FoG + patients present a greater impairment of executive functions, in comparison with those without FoG.

Our data show that the impairment in planning and problem solving (WCST), visual searching (TMT A), and verbal learning (RAVLT) is more pronounced in patients with FoG, while the visuo-constructive functions (ROCF), verbal fluency, delayed recall (both for RAVLT and ROCF), interference (Stroop Test), divided attention, and attention shifting (TMT B and B-A) do not significantly differ between FoG + and FoG- patients. Previous data showed comparable results for Stroop Test performances in patients with and without FoG (Amboni et al., 2008; Tessitore et al., 2012a, b; Vandenbossche et al., 2012). Considering TMT B, while Amboni et al. (2010) found that FoG + patients get worse results compared with patients without FoG, Nantel et al. (2012) did not find differences. In line with previous studies (Amboni et al., 2008; Naismith et al., 2010; Vandenbossche et al., 2012), but not with others (Tessitore et al., 2012a), we observe similar results in verbal fluency performances in both populations. Finally, Tessitore et al. (2012b) found that FoG + patients are impaired in verbal learning but not in visuo-constructive capabilities.

A comprehensive evaluation of executive domains shows that FoG + patients present deficits of planning and a reduced ability to use feedback from the environment and adjust their incorrect behaviors. This evidence is worthy of consideration, as it could provide another point for explaining why during FoG episodes patients seem unable to activate a correct automatic or voluntary gait pattern (Nutt et al., 2011; Vandenbossche et al., 2013). A different aspect concerns the links among FoG occurrence/severity and the cognitive load subtended by the abovementioned functions. Naismith et al. (2010) highlighted a relation between FoG and TMT B. Previous studies showed a relation between cognitive functions and gait (Montero-Odasso et al., 2012), as well as between cognitive functions and FoG (Giladi and Hausdorff, 2006; Spildooren et al., 2010; Janssen et al., 2019): together with the understanding of the effects of the dopaminergic drugs, results from these studies could partially explain why patients with FoG can improve or, conversely, worse the quality of their gait depending on the cognitive load (Muralidharan et al., 2017).

Executive dysfunction in FoG + patients largely relies on overlapping brain networks beyond the sole frontal cortical regions (Amboni et al., 2008; Shine et al., 2013; Brugger et al., 2015). Some interesting data demonstrated that a breakdown in the fronto-parietal attentional control network might be implicated during FoG (Gilat et al., 2018). This network is physiologically recruited to exert top–down control over different contexts (Ptak, 2012). Dysfunctions of these neural circuitries could subtend certain executive deficits that hamper to provide the right top–down control over different internal and external responses, fostering in turn the occurrence of FoG during the parallel processing demands of walking (Gilat et al., 2018). Coherently, task-based functional magnetic resonance imaging studies have shown a reduced activation in cortico-striatal loco-motor pathways and a decrease in functional connectivity between the fronto-parietal attentional control network and the basal ganglia in PD patients with FoG (Shine et al., 2013).

In line with these literature data, our results strengthen the idea that FoG is a “circuitopathy” related to a dysfunctional cortical–subcortical communication (Pozzi et al., 2019). Pozzi et al. (2019) described a cortical–subcortical low frequency synchronization during effective walking and found that the disruption of this cortical–subcortical coupling anticipated and specifically characterized the FoG episodes (Pozzi et al., 2019).

It is notable that, in our FoG + patients, the cognitive dysfunctions do not involve only the executive domain but also the long-term memory. This result strengthens the idea that FoG depends also on cholinergic cortical pathways degeneration, other than on the striatal dopaminergic neuronal loss, thus resembling what happens in condition like dementia (Snijders et al., 2016; Bohnen et al., 2019).

Regarding affective symptoms, previous studies showed a relation between anxiety and FoG occurrence (Giladi and Hausdorff, 2006; Lieberman, 2006). We did not find greater levels of anxiety in FoG + patients; neither the factorial analysis showed a relation between anxiety and FoG. Ehgoetz Martens et al. (2014) found similar results: in this study, the authors observed a greater impact of state anxiety in FoG + patients in comparison with those without FoG only in a specific context, that is walking. Conversely, levels of both state anxiety (i.e., context-dependent anxiety) and trait anxiety (i.e., anxiety as personality feature) did not differ between FoG + and FoG- patients.

Finally, we found that apathy is significantly much more represented in FoG + patients than FoG- patients. Apathy is a disorder of motivation characterized by the lack of spontaneous and goal-directed behaviors that generates a maladaptive state of subjects with respect to the environment.

The cortical–basal ganglia circuit represents the heart of the motivational–emotional system: through inputs and outputs between the striatum and midbrain dopaminergic neurons, information flows from limbic to motor circuits, providing a mechanism by which motivation influences motor decision-making processes (Harsay et al., 2011). Ventral striatum receives prominent inputs from the orbitofrontal cortex (Harsay et al., 2011): both of these structures, together with the anterior cingulate cortex, have been shown to be involved in motivational aspects of behavior (Haber, 2016). Anterior cingulate cortex, through connections with the supplementary motor area (Arienzo et al., 2006), provides the maintenance of the “ongoing” behavior until its completion, reduces conflicts from the environments, stabilizes the action’s performance (Carter et al., 1998), and provides feedback for the guidance of movements (Arienzo et al., 2006).

Previous studies suggest that the presence of apathy and long-term memory deficits is underlined by the disruption of this functional network (Starkstein et al., 1992; Siegel et al., 2014).

To conclude, data from this study suggest that FoG is a paroxysmal condition that results from a chain of events following the alterations of dopaminergic pathways. Specifically, (1) the altered signal generating from putamen and directed to SMA alters gait automaticity; (2) the dopaminergic depletion in the ventral–orbitofrontal cortex pathway reduces the power of the reward system to drive and maintain correct motor behaviors; and finally, (3) disruption of the caudate–prefrontal cortex pathway deteriorates the executive–voluntary system of action control. It follows that during an FoG episode, the motor behavior seems to remain “frozen” in a sort of *limbo* in which both motivational and cognitive systems fail in the attempt to generate a functional, effective outputs for driving the action to move (Ferrazzoli et al., 2018).

This study presents some limitations that have to be acknowledged: first of all, we did not assess FOG-Q in all patients. Indeed, although the most common practical methodologies to screen patients with and without FoG in the clinical setting have been adopted, we were not sure that all patients classified as FoG- would have had the score 0 at the FOG-Q. Therefore, we did not perform correlative analysis between FOG-Q and clinical variables in FOG- patients. This is another limitation of this work, since these data could have had provide relevant results and deepening our knowledge about what differentiate FOG + and FOG- patients in our population.

## Conclusion

The present study provides a new conceptualization of FoG: this phenomenon could be interpreted as consequence of the defects in both the planning and the reward-based guidance of motor behavior. The younger age of FoG + patients at the disease onset suggests a greater pathological involvement of the cortical structures in PD patients who freeze with respect to those without FoG. Therefore, from a behavioral point of view, the presence of FoG could be the manifestation of a “specific phenotype” of PD.

Further studies are needed to better understand the relations linking cognition, motivation, emotions, and FoG and define therapeutic strategies based on this behavioral conceptualization of this phenomenon.

## Data Availability Statement

The datasets generated for this study are available on request to the corresponding author.

## Ethics Statement

The studies involving human participants were reviewed and approved by “Comitato Etico Interaziendale delle Province di Lecco, Como e Sondrio.” The patients/participants provided their written informed consent to participate in this study.

## Author Contributions

PO, DF, and GF contributed to conception and design of the study. PO, VC, MZ, GP, and AG organized the database. RM performed the statistical analysis. PO, DF, and MC wrote the first draft of the manuscript. PO, DF, RM, and MC wrote the sections (Abstract, Introduction, Materials and Methods, Results, Discussion, and Conclusion) of the manuscript. PO, DF, GF, RM, and MC gave substantial contributions to the interpretation of data. All authors contributed to manuscript revision, read, and approved the submitted version.

## Conflict of Interest

GF was employed by company MIRT ParkProject. The remaining authors declare that the research was conducted in the absence of any commercial or financial relationships that could be construed as a potential conflict of interest.
